# OCT macular changes in type 1 ROP following Ranibizumab injections

**DOI:** 10.1186/s40942-025-00664-7

**Published:** 2025-04-17

**Authors:** Rawan Hosny, Jylan Gouda, Tamer A. Macky, Ayman Khattab, Hany Mekkawy, Abdussalam M. Abdullatif

**Affiliations:** Department of Ophthalmology, Kasr AlAiny Hospital, Cairo University, 86 Saraya Street, Al Manial, Cairo, 11956 Egypt

**Keywords:** Retinopathy of prematurity, OCT, Intravitreal Ranibizumab, Macular edema

## Abstract

**Aim:**

To investigate the OCT macular changes in type 1 ROP one month following Ranibizumab injections.

**Methods:**

Preterm infants with type 1 ROP indicated for Ranibizumab injections were included in this study. Handheld OCT imaging was performed at baseline, 1 week, and 1 month post injection. Central full thickness (CFT), inner retinal layer (IRL), and outer retinal layer (ORL) thickness measurements were taken from foveal center and parafoveal region.

**Results:**

24 eyes of 12 infants were included in this study. There were no significant changes in the mean CFT and IRL thickness at 1 month (p = 0.5 and 0.1 respectively). However, there was significant increase in the mean ORL thickness at 1 month (69.9 ± 16, 96.1 ± 25 at baseline and one month respectively, p < 0.001), with differentiation (appearance of IS/OS junction ± ELM) in 55.6% of eyes. Macular edema (ME) was observed in 12 eyes (50%) and was associated with smaller birth weight (p = 0.0290). There was no significant decrease in mean CFT in eyes with ME at 1 month (p = 0.13), with complete resolution in only 6 eyes (50%) during the study period. Regression of plus was associated with lower CFT (1 week and 1 month; p = 0.02 and 0.03, respectively).

**Conclusion:**

Ranibizumab treated eyes in type 1 show ORL thickening and differentiation but with inadequate resolution of ME.

## Introduction

Retinopathy of prematurity (ROP) is one of the leading causes of childhood blindness. It occurs due to arrested development of retinal vasculature. Risk factors include low gestational age and birthweight as well as number of co-morbidities; as sepsis, anemia and the need for O2 support [1]. Current treatment options for type 1 ROP include laser photocoagulation and intravitreal injection (bevacizumab, Ranibizumab and recently aflibercept has been approved by the FDA). [2–5]

Optical coherence tomography (OCT) is a diagnostic imaging tool that provides cross sectional retinal images. The role of OCT in neonates screened for ROP is growing; it detects subclinical findings as cystoid macular edema, retinal layer schisis and epiretinal membranes [6]. This is important, as functional outcome does not always correlate with clinical retinal structural outcome [7].

Infant and preterm maculae have distinct features compared to adults. Infants have thick inner retinal layers compared to thin condensed layers in adult macula; thin outer retinal layers in infants correspond to thick outer layers in adults. [6]

In this study we investigated macular OCT change in Type 1 ROP for a month following intravitreal Ranibizumab injections. To help us better understand the subclinical macular findings in ROP, the effect of the intravitreal Ranibizumab injections on these findings, and their correlation with risk factors.

## Patients and methods

This is a single center prospective interventional study, which included infants diagnosed with type 1 ROP presenting at Cairo University Pediatric Ophthalmology and Strabismology Unit between November 2022 and April 2023. This study followed the tenets of Declaration of Helsinki and the study protocol was revised and approved by ethical committee at Kasr Al Ainy hospital Cairo University with Institutional review board (IRB) number MS-23-2023 and written informed consent was obtained from all the patients’ parents. “Clinical trial number: not applicable.”

### Study population

#### Inclusion criteria

The study included infants with Type 1 ROP diagnosis (any stage in zone I associated with plus disease, stage 2 or 3 in zone II associated with plus disease, stage 3 in zone I or aggressive posterior ROP)—both treatment naïve and cases requiring re-injection. Exclusion criteria: (1) eyes with media opacity hindering adequate OCT acquisition: e.g., congenital cataract, (2) congenital glaucoma, (3) anterior segment anomalies, (4) posterior segment anomalies: e.g., choroidal coloboma and morning glory anomaly, (5) retinal detachment associated with ROP (Stages 4 and 5), and (6) any pathology of the macula or the optic nerve in the included eyes.

### Baseline demographics and clinical data

- Preterm gestational age (GA), sex, NICU admission duration, associated comorbidities, birth weight (BW), presence of twin sibling and age at time of diagnosis [post menstrual age (PMA)].
- Infants were examined using binocular indirect ophthalmoscopy, after placement of lid speculum using scleral indentation and topical anesthesia. ROP examination data (zone, stage, plus) was documented on each visit.

### Retinal imaging

- The infants were imaged by both OCT macula (Handheld OCT iVue: HH OCT. RTVue RT-100, Optovue Inc., Fermont, CA) and RetCam (Clarity Medical Systems, Inc., Pleasanton, CA, USA). Infants were imaged after falling asleep, without sedation. Lids were opened gently by parents or a nurse. A dextrose dipped pacifier may be used to calm baby down. Occasionally, the mother may hold the baby and imaging was performed while mother sat on a chair, to reduce infant stress.
- Macular scans were taken and optimized for obtaining a single high-quality scan at the fovea. Identification of the foveal center was achieved by detecting the deepest point in the central retina. Foveal and parafoveal thickness was obtained using caliper (manually)—cross-line image, with inner retinal layers (IRL) and outer retinal layers (ORL) measured. Inner retinal layers: from internal limiting membrane till the outer border of the inner nuclear layer, outer retinal layers: from the inner border of the outer plexiform layer to the outer border of retinal pigment epithelium (RPE).
- Central foveal thickness (CFT) from internal limiting membrane to RPE. Parafoveal measurements were taken 1000 μm from either side of the center of the fovea. The fovea-parafoveal ratio (FP) was obtained by dividing CFT by the average of 2 parafoveal measurements. An FP > 1 suggest a bulging fovea, while < 1 suggests the presence of a pit. Intraretinal hypo-reflected spaces distorting the foveal contour with vertical hyperreflective intervening septae were defined as Macular Edema (ME).

### Intravitreal Ranibizumab injections

Injections were carried out within 2 days of diagnosis. Ranibizumab 0.25 mg/0.025 ml was injected under complete sterile conditions in operating room using topical anesthesia, 1 mm from limbus on the temporal side.

### Follow up visits

Clinical examination using binocular indirect ophthalmoscope was done first day and then every week following the Ranibizumab injections to follow up ROP status. RetCam and OCT images were taken one week and one-month post injection. *Regressed ROP* is a post injection status in which there is incomplete regression of the plus disease but with more arterial tortuosity OR more venous dilatation than normal. *No plus ROP:* is a post injection status in which there is complete regression of the plus disease.

### Outcome measures

(A) Primary outcomes:
  1. Central foveal thickness, inner retinal and outer retinal layers thickness before and after Ranibizumab injection for type 1 ROP.
  2. Macular edema: its prevalence and one month response to the Ranibizumab injections.

(B) Secondary outcomes:
  1. Correlations: between baseline and 1 month OCT thickness and BW, GA, PMA and NICU duration.
  2. Relationship between OCT findings and each Plus disease, and preterm systemic status.
  3. Foveal architectural changes following Ranibizumab injection

### Statistical methods

Data were coded and entered using the statistical package for the Social Sciences (SPSS) version 29 (IBM Corp., Armonk, NY, USA). Data was summarized using mean, standard deviation, median, minimum and maximum in quantitative data and using frequency (count) and relative frequency (percentage) for categorical data. Comparisons between quantitative variables were done using the non- parametric Mann–Whitney test when comparing two groups, if more Friedman test was used for repeated measures (non-parametric ANOVA). The Kruskal–Wallis test was used for multiple group (non- repeated measures) comparison of continuous data. For comparing categorical data, Fisher exact test was used. Correlations between quantitative variables were done using Spearman correlation coefficient. The Shapiro–Wilk test was used to test for normality. p-values less than 0.05 were considered as statistically significant.

## Results

Twenty-four eyes of 12 infants fulfilling our inclusion/exclusion criteria were enrolled in our study. All infants had bilateral ROP, but OCT image measurements of two eyes could not be obtained at baseline, one due to presence of pre-macular hemorrhage and the other due to poor quality, thus only 22 eyes were analyzed at baseline. Later, one infant was admitted to the ER due to complicated inguinal hernia and missed the one month follow up. Additionally, at 1 month one eye of two infants could not be obtained due to difficulties with OCT imaging. Therefore, only 18 eyes had OCT images analyzed at the 1-month visit. Each infant received one Ranibizumab injection.

### Infants demographic features and systemic status

The male: female ratio was 1:1, with a mean GA of 29.75 (range 26–33) weeks, and a mean BW of 1486.67 (range 1100–2000) grams. The mean PMA of type 1 diagnosis was 36.7 ± 3.15 weeks (32–42 weeks) while the mean PMA a A-ROP diagnosis was 36.3 ± 2.83 weeks PMA (32–40 weeks). All infants were admitted for a mean of 35.5 days (12–70) in NICU, 9 (75%) received oxygen support, and 7 (58.3%) received blood transfusion of which 2 (16.6%) had seizures. One infant had hydrocephalus and one had intracranial hemorrhage. One infant died in March 2023 (after completion of imaging). Six infants had a twin sibling and one was part of triplets—all siblings had ROP not indicated for treatment or were deceased.

### ROP features during the study period

Baseline: nine eyes (37.5%) had AROP, and 15 had Type 1 ROP: 11 (45.83%) stage 3, and 4 (16.67%) stage 2. The ROP was in zone II in 19 eyes (79.17%) and in zone I in 5 eyes (20.83%) and all had plus disease.

One week post injection: 4 eyes (16.67%) had stage 3, 10 eyes (41.67%) had stage 2, 4 eyes (16.67%) had stage 1, and 6 eyes (25%) had stage 0. The ROP was in zone II in 22 eyes (91.67%) and zone I in 2 eyes (8.3%). Twenty eyes (83.3%) had regressed plus disease and 4 (16.67%) had no plus disease.

One month post injection: 7 eyes (29.17%) had stage 2, 5 eyes (20.83%) had stage 1, and 12 eyes (50%) had stage 0. The ROP was in zone II in 23 eyes (95.83%) and zone III in one eye (4.17%). Twenty eyes (83.3%) had no plus disease, 2 eyes had regressed plus (8.3%), and 1 child had worsened plus status after initial regression (Table 1).

**Table 1:** Final clinical outcomes

ROP characteristics (1 month post IVI)	
Stage 0, No. of eyes (%)	12, (50%)
Stage 1, No. of eyes (%)	5, (20.83%)
Stage 2, No. of eyes (%)	7, (29.17%)
Zone II, No. of eyes (%)	23, (95.83%)
Zone III, No. of eyes (%)	1, (4.17%)
Regressed plus, No. of eyes (%)	2, (8.3%)
No plus, No. of eyes (%)	20, (83.3%)
Pre-plus/plus, No. of eyes (%)	2, (8.3%)

### OCT analysis

#### Retinal thickness

**Fig. 1:**
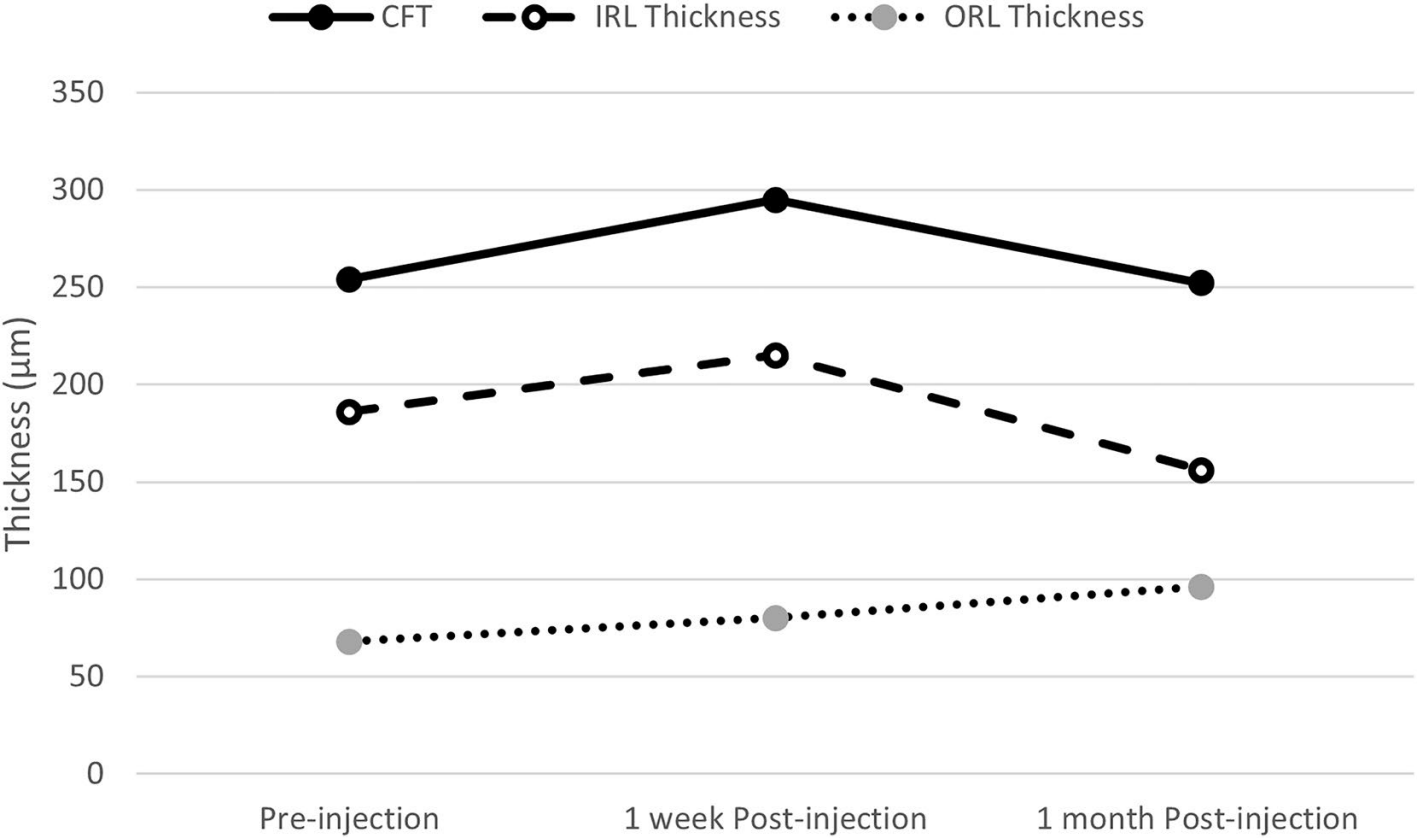
Mean central foveal thickness (CFT), inner retinal layer thickness (IRL), and outer layer thickness (ORL) curve over the consecutive follow up periods at baseline, one week and one month post-injection

The mean CFT, IRL thickness, ORL thickness, and FP ratio during the study period visits are summarized in Table 1 and Fig. 1. At one month there were no significant changes in CFT, IRL thickness, FP ratio. However, there was a significant increase in ORL thickness. There were also no significant changes in parafoveal retinal thickness (p = 0.1), or parafoveal IRL thickness (p = 0.6), but a significant increase in parafoveal ORL (p < 0.0001) (Table 2).

**Table 2:** OCT measurements during the study period

	Baseline (n = 22)	One week (n = 22)	One Month (n = 18)	p value
Central foveal thickness µm ± SD (range)	`250.8 ± 104.3 (112—461)	268.1 ± 147.1 (115—571)	252.1 ± 132.2 (128—520)	0.54
Outer retinal layers thickness µm ± SD (range)	69.95 ± 16.47 (48–104)	79.41 ± 22.11 (48—128)	96.17 ± 25.34 (55—133)	0.0006
Inner retinal layer thickness µm ± SD (Range)	180.9 ± 103 (52—388)	188.7 ± 147.8 (21—483)	155.9 ± 146.2 (17- 42)	0.17
Fovea-parafoveal ratio	0.85 ± 0.25 (0.46–1.28)	0.84 ± 0.32 (0.4–1.49)	0.79 ± 0.29 (0.46–1.29)	0.2

Correlation analysis was done between retinal thickness and GA, PMA, BW, and NICU duration. At baseline, there were negative correlation between BW and each of CFT (spearman r = − 0.43 and p = 0.04) and IRL (spearman r = − 0.45 and p = 0.03). Also, a positive correlation was found between ORL thickness and age at time of imaging (r = 0.48, p = 0.02). Retinal thickness changes at 1 month showed a negative correlation between the ORL change and age of presentation (r = − 0.56, p = 0.01). No other correlations were found between retinal thickness and GA, PMA, BW, and NICU duration at baseline or changes occurred at 1 month.

There was no statistically significant relationship between baseline ROP staging and foveal measurements. However, regression of plus was associated with lower CFT (p = 0.02 post 1 week and 0.03 post 1 month).

### Macular edema

#### (A) Incidence

ME was found in 6 infants (12 eyes, 50%) 10 at baseline and 2 appeared at 1 week. There was significant association between ME and smaller BW (p = 0.02). However, there were no association found between ME and each of GA (p = 0.6), PMA (p = 0.8), ROP staging (p = 0.09) or time spent in NICU (p = 0.2). Staging of eyes with ME was: five eyes were stage 3, three stage 2 and two AROP while all eyes had plus and zone II disease. All infants had history of blood transfusion (p = 0.006), one had hydrocephalus and another intracranial hemorrhage.

#### (B) Ranibizumab response

**Fig. 2:**
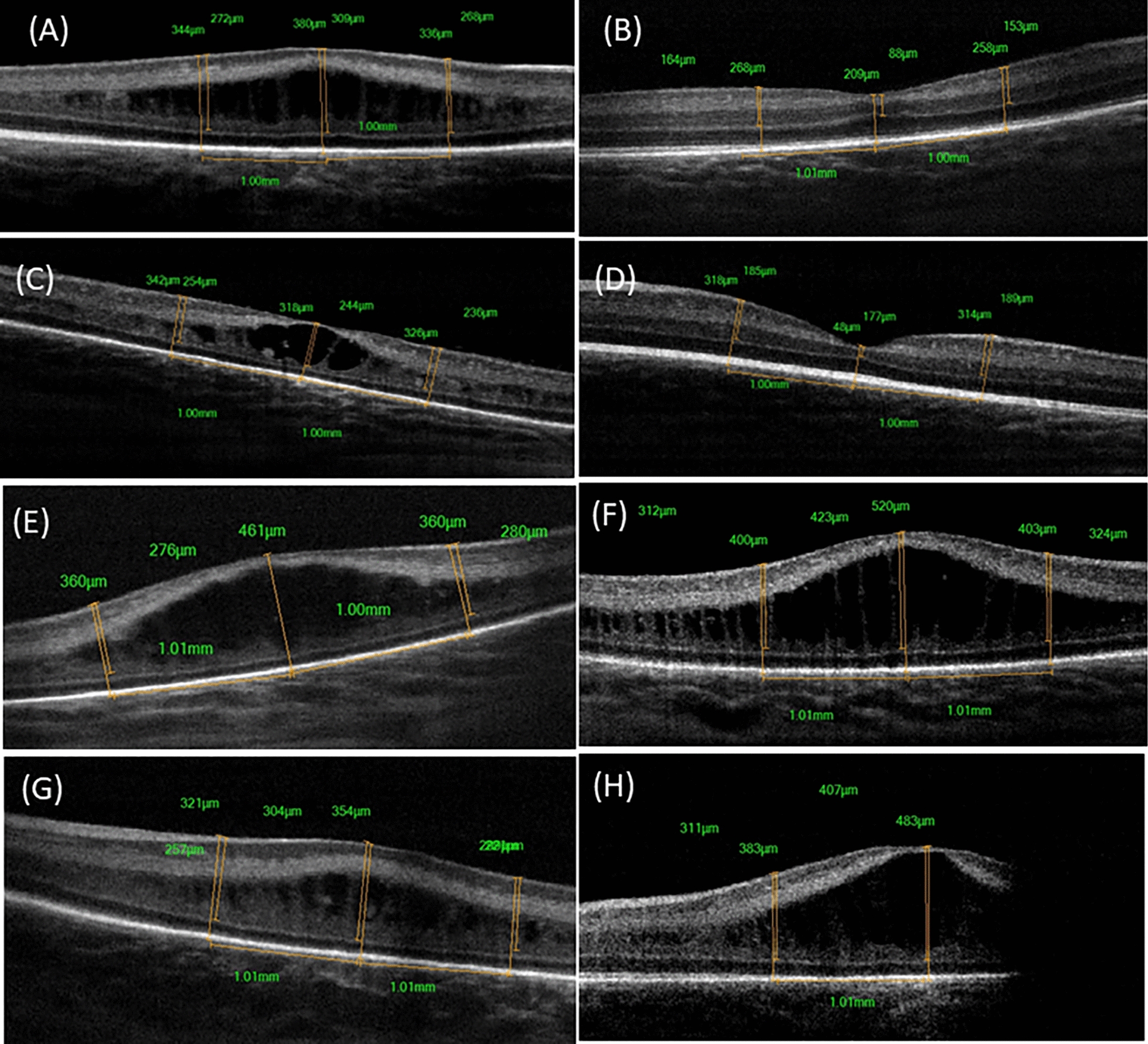
Spectral domain optical coherence tomography (SD-OCT) scans of left eye of a male infant born at age of 28 weeks with 1200 gm admitted in NICU for 70 days and presented at age of 36 weeks with Type 1 ROP (ROP zone II, stage 3, and plus disease) (**A**) OCT image at time of presentation with macular edema (ME) and CFT 380 µm and **B** OCT image at one month showing resolution of ME (CFT 209 µm) with ORL thickening and differentiation but with delayed IRL differentiation. OCT images of right eye of a female infant born at age of 28 weeks with 1100 gm admitted in NICU for 52 days on ventilator and presented at age of 38 weeks with Type 1 ROP (ROP zone II, stage 3, and plus disease), **C** OCT image at time of presentation with macular edema (ME) and CFT 318 µm and **D** OCT image at one month showing resolution of ME (CFT 177 µm) with ORL thickening and appearance of IS/OS junction and IRL differentiation. OCT images of left eye of a female infant born at age of 28 weeks with 1800 gm admitted in NICU for 12 days and presented at age of 33 weeks with Type 1 ROP (ROP zone II, stage 2, and plus disease), **E** OCT image at time of presentation with macular edema (ME) and CFT 461 µm and **F** OCT image at one month showing worsening of ME (CFT 520 µm) with poor retinal layer differentiation. Similarly, OCT images of right eye of a female infant born at age of 32 weeks with 1140 gm admitted in NICU for 46 days with history of sepsis, presented at age of 38 weeks with aggressive ROP, **G** OCT image at time of presentation with macular edema (ME) and CFT 354 µm and **H** OCT image at one month showing worsening of ME (CFT 483 µm) with poor retinal layer differentiation

**Fig. 3:**
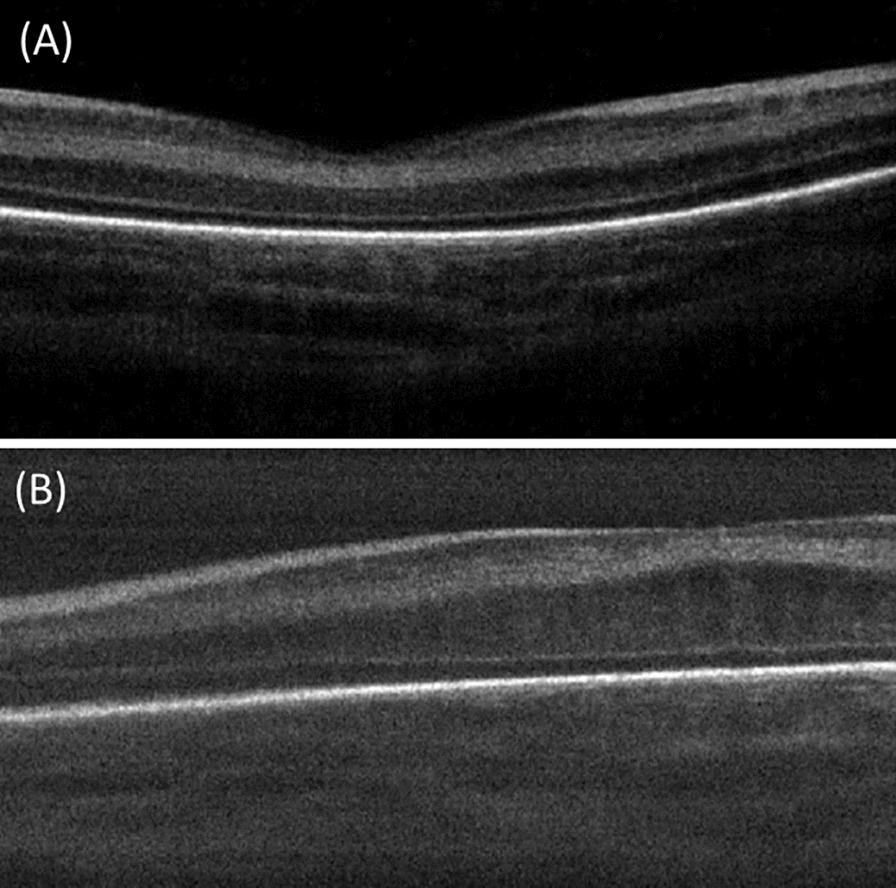
Spectral domain optical coherence tomography (SD-OCT) scans of right eye of a male infant born at age of 28 weeks with 1500 gm admitted in NICU for 25 days and presented at age of 32 weeks with Type 1 ROP (ROP zone II, stage 3, and plus disease): **A** OCT image at time of presentation showed no macular edema (ME) and CFT 196 µm, **B** OCT image at one month showing development of ME which has not been present pre injection (CFT 307 µm) with poor retinal layer differentiation

CFT measurements in patients with ME did not decrease significantly from baseline to post 1 month (p = 0.38). During the study 1-month period, ME completely resolved in six eyes (50%) presenting with ME. Two eyes (15%) resolved at one week and four eyes (33%) at one month—2 eyes of which initially worsened at 1 week (Fig. 2A–D). Thus, six eyes had edema present at the final image compared to 12 eyes at baseline (p = 0.58). The mean PMA at which ME resolved was 40.3 ± 1.36 weeks. Three patients (6 eyes, 50%) had worsened edema 1 month following injection however, the worsened edema was not associated with worsened ROP staging (Fig. 2E–H). One child of those infants was systemically worsened (had a respiratory tract infection and loss of weight), the second was generally stable. The third infant (2 eyes) did not present initially with edema but developed macular edema post injection (Fig. 3). This infant, however, died in March 2023.

### Foveal architectural

**Fig. 4:**
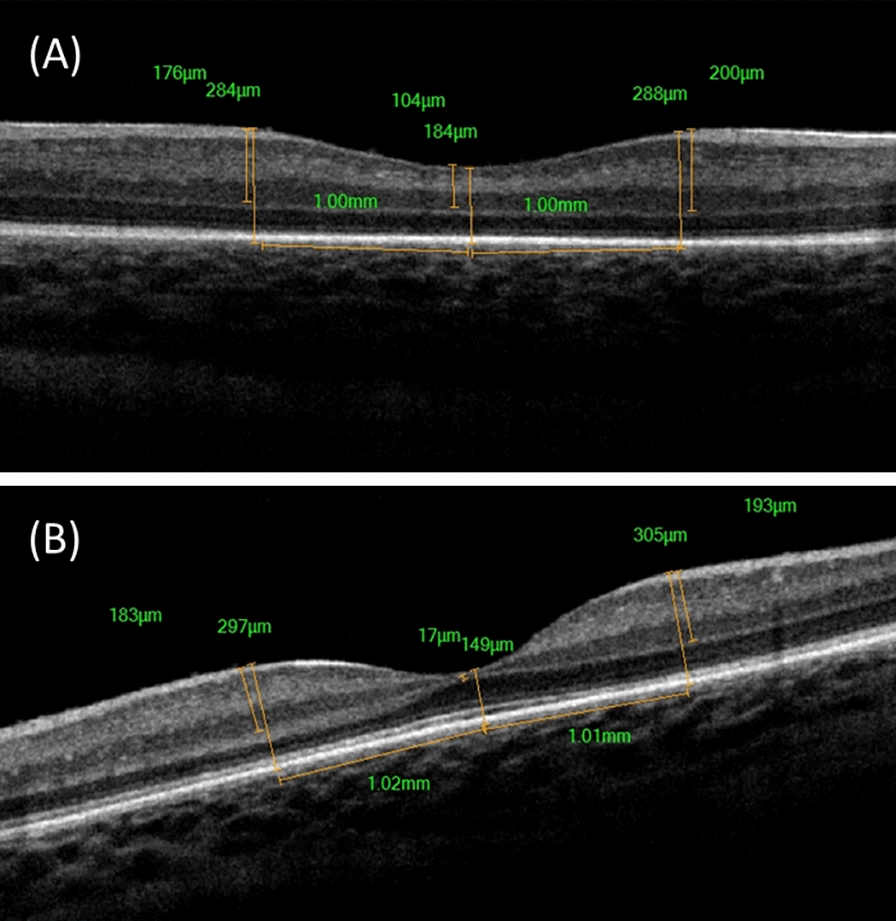
Spectral domain optical coherence tomography (SD-OCT) scans of left eye of a male infant born at age of 30 weeks with 1370 gm admitted in NICU for 40 days and presented at age of 39 weeks with aggressive ROP: **A** OCT image at time of presentation, **B** OCT image at one month showing deepening of foveal pit and thickening and differentiation of outer retinal layers (appearance of IS/OS junction and ELM)

Foveal architectural changes were noticed following Ranibizumab injection. The mean age of patients who showed ORL differentiation following Ranibizumab injection was 40.3 weeks PMA with eyes showing ORL differentiation being older (42 ± 2.5 weeks PMA) than without (38.25 ± 2.7 weeks PMA) (p = 0.017). The foveal pit in infants not presenting with edema becomes deeper with progressive thinning of the inner retinal layers. Thickening and differentiation of outer retinal layers (i.e., appearance of IS/OS junction ± appearance of ELM) is noticed as well in 10/18 eyes (55.6%) which completed post 1 month imaging (Fig. 4). All eyes with persistent edema at post 1 month imaging showed poor ORL differentiation. The 10 eyes with ORL differentiation have thicker ORL (mean thickness 111.3 ± 21.73 μm) compared to the 8 eyes with delayed ORL differentiation (mean 77.25 ± 14.71 μm) (p = 0.003).

## Discussion

Retinopathy of prematurity is one of the leading causes of childhood blindness. Long-term follow up of ROP treated eyes showed that good structural outcome (i.e., normal retina) was not always associated with good functional outcome [7–9]. Better understanding of the subclinical changes with the help of OCT might lead to better treatment options. In our study we investigated the foveal and parafoveal OCT changes associated with Ranibizumab injection for type 1 ROP. To our knowledge, this is the first prospective study documenting these changes, with only 2 retrospective studied reporting changes following bevacizumab [10], and Ranibizumab [11].

Changes in macular layers’ thicknesses, structures, and architectures in preterm babies is not fully understood, and various studies have shown inconsistent factors interplay. These reported factors are the normal developmental changes, the preterm general condition, GA, PMA, presence or absence of ROP, ROP stage, ME, VEGF vitreous load, and the use of different types of anti-VEGFs. In our study, we have observed some of these factors to be significant and while other factors showed trends that were not significant. In addition, our findings were not all consistent with previous reports, due to the small number of eyes and the limited studies in the literature on the subject.

In our study, CFT showed non-significant increase at 1 week, and non-significant decreased at 1 month following Ranibizumab injections. Erol et al. [11] demonstrated similar results with the mean CFT increased at 1 week following intravitreal Ranibizumab injections and then decreased again at 2 months. Vogel et at [12] demonstrated that CFT following bevacizumab injection steadily increased—which is contradictory to our results. They did, however, exclude imaging showing ME because of the significant distortion of foveal architecture. On the other hand, we had a statistically significant thickening of ORL similar to Vogel et al. [12] study where bevacizumab treatment was associated with a more rapid ORL thickening and development. We didn’t find a correlation between CFT or ORL thickness and ROP stage, but Maldonado et al. [13] reported that the median CFT and IRL thickness were higher in eyes with stage 3 ROP.

IRL development appears to be an independent process. Persistence of one or more layers is a constant feature of the fovea of premature infants and the rate of decrease in IRL thickness is not associated with the rate of development of ORL. We found that a significant thinning of IRL occurred between 1 week and 1 month following IVI. Interestingly, a study by Maldonado [14] mentioned the IRL changes occurring from 31 to 45 weeks PMA in four infants without macular edema. Progressive decrease in IRL thickness with progressive increase in parafoveal IRL thickness and thus lower FP with increasing age was found. Whether the significant drop in IRL thickness is related to age of infant or to effect of anti-VEGF is debatable. Their study also excluded type 1 ROP patients, the pathology of which could possibly delay foveal development compared to premature infants without/with milder stages of ROP. However, as with CFT and ORL, we didn’t find a correlation between IRL thickness and ROP stage, similar to Dubis et al. [15]

ME incidence has been reported in many studies in premature infants with and without ROP to occur in 16% to 88.9% [11]. We found significant associated between ME and lower BW but not GA. However, these associations were inconsistent in among many studies [14–16]. Subclinical macular edema has been reported to occur in all stages of ROP and its incidence does not appear to be correlated with ROP staging. [15, 16]. Similar to CFT, ORL and IRL thicknesses, we didn’t find significant relation between ME and ROP stage, while other reports found increased incidence of edema with higher staging. [17, 18]

This observation raised questions regarding the pathological nature of ME in premature infants. It has been hypothesized that increased VEGF levels contribute to developing of ME in premature infants and thus, as in adults with macular edema secondary to diabetic retinopathy or vein occlusion, anti-VEGFs should lead to effective resolution of ME in ROP [13]. The non-significant reduction in the mean CFT in eyes with ME in our study with only a 50% resolution at 1 month following intravitreal Ranibizumab raises more questions to the role of VEGFs in its development. It is our observation that ME is anti-VEGF independent. One month after anti-VEGF injections, half of the ME resolved and half worsened with overall mean thickness remained unchanged is strong indicator that it is anti-VEGF independent. Mechanical factors and other neurohormonal factors are thought to play a role in the development of ME in ROP [15]. It has been also found that RPE density is lower in premature infants, which may also contribute to development of ME. [17] Vascular congestion from plus disease may contribute to its development as well. [13] In our study, plus disease was associated with thicker CFT (p = 0.02 and 0.037).

All infants with ME in our study had fluid exclusively in the INL. ME in adults, on the other hand, presents with fluid accumulation in multiple retinal layers [18]. There is evidence of both extra and intracellular fluid accumulation of Muller cells in adults with ME [19]. ME in premature infants may thus be secondary to Muller cell swelling without extracellular leak. Extracellular leak in adults is thought to be secondary to ischemia leading to Muller cell death and disruption—this mechanism however seems very unlikely in the premature eye.

ME of prematurity could also be thought of as a physiological rather than pathological process, as it has been found in premature infants with no ROP [20] and it has been observed to resolve spontaneously [21]. It does not, however, occur in healthy full-term infants [22], which lead to the hypothesis that it may be a transient stage in retinal development. It may represent rapid development of foveal cones during this time frame. [13]

The first in vivo study to evaluate the maturation of the fovea described the following; as the premature eye grows, thickness and number of IRLs decreased, foveal pit deepened, while the ORL thickened and became more differentiated. The IRL and ORL development appeared to be independent on one another. IRL thickness decrease was delayed in infants with ME, while the ORL thickening and differentiation seemed to progress normally [14]. Our results, however, show that all eyes with persistent edema at post 1 month imaging showed poor ORL differentiation.

The ORL development seem to be more crucial in determining visual outcome. Apart from thickening, differentiation of photoreceptor sublayers has been seen to develop as the infant grows. Both ELM and IS/OS junction were not seen in all infants 31 to 42 weeks PMA and their appearance was variable between subjects, appearing for example in three preterm infants between 43 and 48 weeks PMA [14]. Vogel et al. [12] reported that the IS/OS junction was seen peripherally and developed centripetally, eventually reaching the center by 41 to 52 weeks PMA. In 55.6% of eyes completing the post 1 month imaging (10/18) there was ORL differentiation, which was noted also to appear in a centripetal fashion. There was a positive correlation with both age at time of imaging and ORL thickness, mean age at time of imaging being 42 weeks. The differentiation at a younger age may be attributed to the anti-VEGF treatment, as noted also by Vogel et al. [12]. Six of less differentiated eyes had ME, however the delay may be due to younger age at time of imaging rather than the effect of edema itself.

Preterm ROP infants are imaged without sedation and the imaging process is very lengthy; waiting for the non-sedated infant to fall asleep, taking breaks when it starts waking up and searching for the macula in a dilated pupil without fixation make imaging in those children a big challenge. Many of these infants are systemically unwell and thus cannot spend much time of the NICU. Also, they may skip follow up OCT due to poor health condition. This limits the number of infants included in our study. Moreover, our study is limited by the fact that the iVue does not have a fast-track system; images could not be repeated from the exact site. The short follow up duration is mainly due to the fact that as an infant gets older, the more difficult it is to obtain OCT images without sedation/anesthesia, the risks of which outweigh the benefits of the OCT imaging. Anatomical outcome was assessed only, as functional outcome is very difficult to assess in the patient population.

## Conclusion

In conclusion Ranibizumab treated eyes show ORL thickening and differentiation with inadequate ME resolution in these eyes. This shows that macular changes in preterm infants with Type 1 ROP are affected by an interplay of multiple involved factors rather than VEGFs dependent etiology. It is certain that a larger study size is essential to better understand the macular changes in preterm infants and the risk factors involved.

## Data Availability

The data that support the findings of this study are not publicly available due to their containing information that could compromise the privacy of research participants but are available from the corresponding author R.H.
